# Activation of caspase-dependent apoptosis by intracellular delivery of cytochrome c-based nanoparticles

**DOI:** 10.1186/s12951-014-0033-9

**Published:** 2014-09-02

**Authors:** Moraima Morales-Cruz, Cindy M Figueroa, Tania González-Robles, Yamixa Delgado, Anna Molina, Jessica Méndez, Myraida Morales, Kai Griebenow

**Affiliations:** 1 Departments of Chemistry, University of Puerto Rico, Río Piedras Campus, San Juan 00931, PR, USA; 2 Department of Biology, University of Puerto Rico, Río Piedras Campus, San Juan 00931, PR, USA; 3 Department of Graduate Studies, University of Puerto Rico, Río Piedras Campus, Río Piedras Campus, San Juan 00931, PR, USA

**Keywords:** Drug delivery, Protein nanoparticles, PLGA, Passive targeting, Triggered release

## Abstract

**Background:**

Cytochrome c is an essential mediator of apoptosis when it is released from the mitochondria to the cytoplasm. This process normally takes place in response to DNA damage, but in many cancer cells (i.e., cancer stem cells) it is disabled due to various mechanisms. However, it has been demonstrated that the targeted delivery of Cytochrome c directly to the cytoplasm of cancer cells selective initiates apoptosis in many cancer cells. In this work we designed a novel nano-sized smart Cytochrome c drug delivery system to induce apoptosis in cancer cells upon delivery.

**Results:**

Cytochrome c was precipitated with a solvent-displacement method to obtain protein nanoparticles. The size of the Cytochrome c nanoparticles obtained was 100-300 nm in diameter depending on the conditions used, indicating good potential to passively target tumors by the Enhanced Permeability and Retention effect. The surface of Cytochrome c nanoparticles was decorated with poly (lactic-co-glycolic) acid-SH via the linker succinimidyl 3-(2-pyridyldithio) propionate to prevent premature dissolution during delivery. The linker connecting the polymer to the protein nanoparticle contained a disulfide bond thus allowing polymer shedding and subsequent Cytochrome c release under intracellular reducing conditions. A cell-free caspase-3 assay revealed more than 80% of relative caspase activation by Cytochrome c after nanoprecipitation and polymer modification when compared to native Cytochrome c. Incubation of HeLa cells with the Cytochrome c based-nanoparticles showed significant reduction in cell viability after 6 hours while native Cytochrome c showed none. Confocal microscopy confirmed the induction of apoptosis in HeLa cells when they were stained with 4’,6-diamidino-2-phenylindole and propidium iodide after incubation with the Cytochrome c-based nanoparticles.

**Conclusions:**

Our results demonstrate that the coating with a hydrophobic polymer stabilizes Cytochrome c nanoparticles allowing for their delivery to the cytoplasm of target cells. After smart release of Cytochrome c into the cytoplasm, it induced programmed cell death.

## Background

Cytotoxic drugs (such as, cis- and carboplatin, doxorubicin, 5-fluorouracil, gemcitabine, paclitaxel, out of a list of over 50) are still commonly used in chemotherapy to stop cancer cells from multiplying and dispersing. These drugs affect all growing cells, but since most normal cells do not divide as often as cancer cells they are proportionately less affected. Unfortunately, the lack of tumor specificity of these cytotoxins produces unwanted and often severe and dangerous side effects. One approach to overcome this is the development of nano-sized drug delivery systems (DDS), which have been shown to increase the drug accumulation in tumors *via* passive targeting, i.e., through the enhanced permeation and retention (EPR) effect [1]-[3]. Two types of nano-sized DDS, liposomes and albumin nanoparticles (NPs), i.e. Doxil® and Abraxane®, respectively, have caused notable improvements in the therapeutic efficacy of anticancer agents [4]. However, also these therapeutics still display significant side effects and only prolong life marginally. The lack of success in improving overall survival in many solid tumors and the still significant side effects of even the second generation drugs make it imperative to further develop and refine such DDS.

All currently US FDA approved chemotherapeutic DDS and most that are in current clinical trials or *in vivo* studies still employ traditional cytotoxic drugs as their therapeutic agents. Many of these chemotherapeutic agents (e.g., alkylating agents and antimetabolites) produce DNA damage which leads to p53-dependent apoptosis [5]. Indeed, the loss of the p53 tumor suppressor pathway contributes to the development of most human cancers [6]. Thus, inactivation of the apoptotic response provides an attractive explanation for the poor responsiveness of p53 mutant tumors to many traditional anticancer agents. Such limitations have spurred efforts to identify new and more effective chemotherapeutic agents that act independently of the p53 pathway. The high selectivity and low toxicity of many proteins make them attractive substitutes of cytotoxic drug. For example, Cytochrome c (Cyt c) is an important mediator of apoptosis when it is released from the mitochondria to the cytoplasm [7],[8]. This process normally takes place in response to DNA damage, but in many cancer cells it is inhibited (most likely due to inactivation of the upstream components of the signaling pathway(s) that activates the Cyt c release, such as the p53 pathway). The targeted delivery of Cyt c directly to the cytoplasm could selectively initiate apoptosis in most cancer cell by circumventing inactivation of apoptosis due to damage to upstream events.

However, protein drugs also do have significant drawbacks, primarily related to their limited physical and chemical stability during storage and after administration [9]-[11]. Also, most proteins including Cyt c cannot cross lipid bilayer membranes [12]. This makes it necessary to develop methods allowing for the intracellular delivery of sufficient amounts of Cyt c to induce apoptosis in the target cells. The development of nanosized carriers for protein therapeutics allows maintaining protein stability and controlling release properties but having a sufficiently high protein loading remains challenging [11],[13]. Recently our research group has been developing strategies for the intracellular delivery of Cyt c using nanosized DDS [14],[15]. We demonstrated that Cyt c delivered from a smart nanosized system could potentially be an effective chemotherapeutic agent to treat cancer, but also recognized that the delivery system used needed improvement to achieve a better efficiency. Specifically, we demonstrated that mesoporous silica nanoparticles (MSN) could be utilized as efficient carrier for the delivery of apoptosis-inducing Cyt c [15]. However, Cyt c-MSN conjugates were not able to induce cell death in HeLa cells during the first 48 h. They showed 45% cell viability after 72 h at a Cyt c concentration of 37.5 μg/ml. It was not possible to increment the Cyt c concentration since that resulted in a toxic concentration of MSN (>100 μg/ml) and the particles were already loaded to the maximum with the protein. This demonstrates fundamental limits of such drug-loaded systems in practical applications. Ideally, to avoid such limitations, the drug should itself form the DDS. Consequently, we evolved a delivery system in which the core consisted of Cyt c NPs and thus the drug itself.

**Figure 1:**
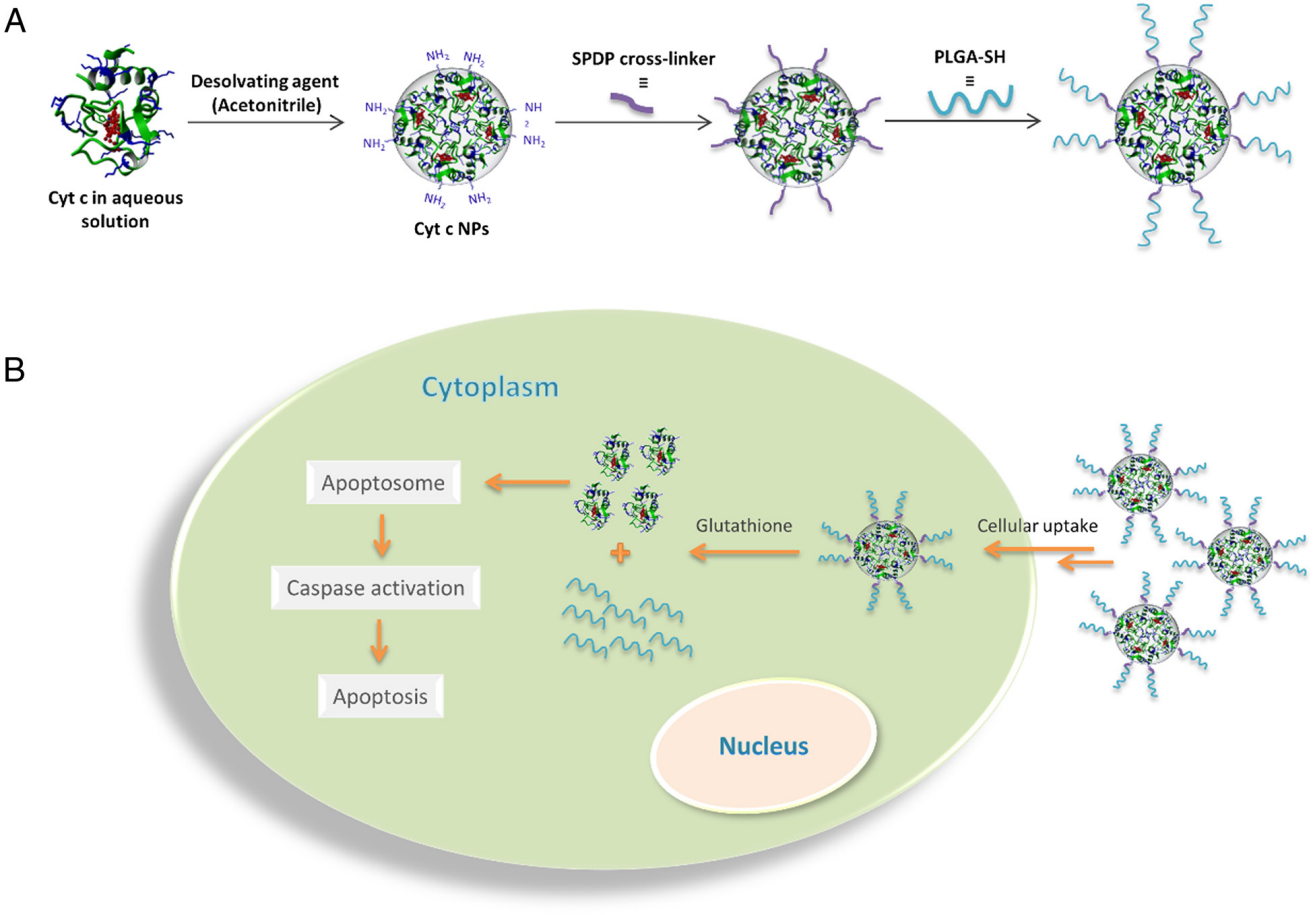
**Schematic representation of the synthesis and application of Cyt c-based NPs. A)** Protein nanoprecipitation followed by the nanoparticles surface modification with PLGA. **B)** Intracellular stimulus-responsive Cyt c release.

Production of Cyt c NPs by a solvent displacement method is a straight-forward process (i.e., by solvent-induced nanoprecipitation) [16]. However, such NPs would simply dissolve when injected into the blood stream and thus need to be stabilized for application, e.g., by applying high temperature and/or chemical cross-linkers [17]. NPs, which were stabilized via a chemical cross-linker agent with thiol-cleavability, have been successfully used for triggered drug release mediated by the reducing environment inside the cell [18]. In this work we tested a new method for stabilizing Cyt c NPs by covalently coating them with the hydrophobic polymer poly (lactic-co-glycolic) acid (PLGA). PLGA, a biocompatible, biodegradable, and non-toxic polymer, has been intensely studied in the field of DDS and has received FDA approval for various applications including drug delivery [19],[20]. Our idea was to coat the Cyt c NPs with PLGA using a hetero-bifunctional linker, such as succinimidyl 3-(2-pyridyldithio) propionate (SPDP), that includes a disulfide bond to be able to shed the polymer shell in the reducing environment of the cell (Figure 1A). Afterwards the Cyt c nanoparticle should dissolve in the cell thus inducing apoptosis (Figure 1B). In this work we demonstrate the potential of the designed anti-tumor Cyt c nanoparticle in cancer treatment.

## Results and discussion

### Nanoprecipitation of Cyt c

Cyt c, an apoptosis-initiating protein, could potentially be used to target and specifically destroy cancer cells if delivered to their cytoplasm. Protein-based nanomaterials have been studied as carriers of anti-cancer drugs because of their biodegradability, low toxicity, and multiple modification capacity [21]. It would be ideal to use the protein as both, nanosized delivery device and drug, simultaneously. To test this concept, in this study we generated and tested Cyt c-based NPs to kill cancer cells by inducing the apoptosis.

**Figure 2:**
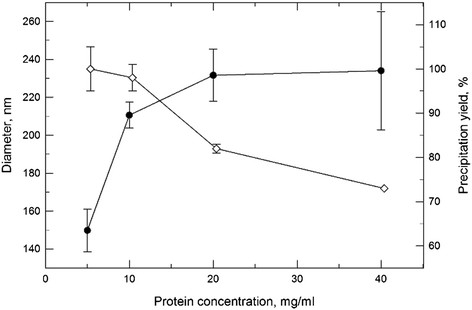
**Effect of the protein concentration during Cyt c nanoprecipitation.** The particle diameter (─●─) and precipitation yield (─◊─) were determined for different protein concentration.

Cyt c nanoparticles were obtained by a solvent displacement method shown to induce a reversible nanoprecipitation of proteins without compromising their frequently fragile structure and function [14]. In a previous study we found that acetonitrile was a useful organic solvent for obtaining Cyt c nanoprecipitates when it was added in at least 4-fold excess to the aqueous protein solution [14]. In this work we optimized this procedure for obtaining Cyt c nanoparticles and tested the effect of protein concentration on Cyt c precipitation and stability. While no buffer-insoluble aggregates were formed regardless of the protein concentration (data not shown), the precipitation yield decreased at higher protein concentrations (Figure 2). In agreement with our previous work for other proteins [14], we also found that at increasing protein concentration under otherwise constant conditions the particle size increased (Figure 2). We selected a Cyt c concentration of 5 mg/ml for further experiments since it had the highest precipitation efficiency (100%) and smallest particle diameter (around 150 nm). Note that a particle diameter of less than 400 nm should result in particles with good passive delivery properties [2],[22],[23].

**Figure 3:**
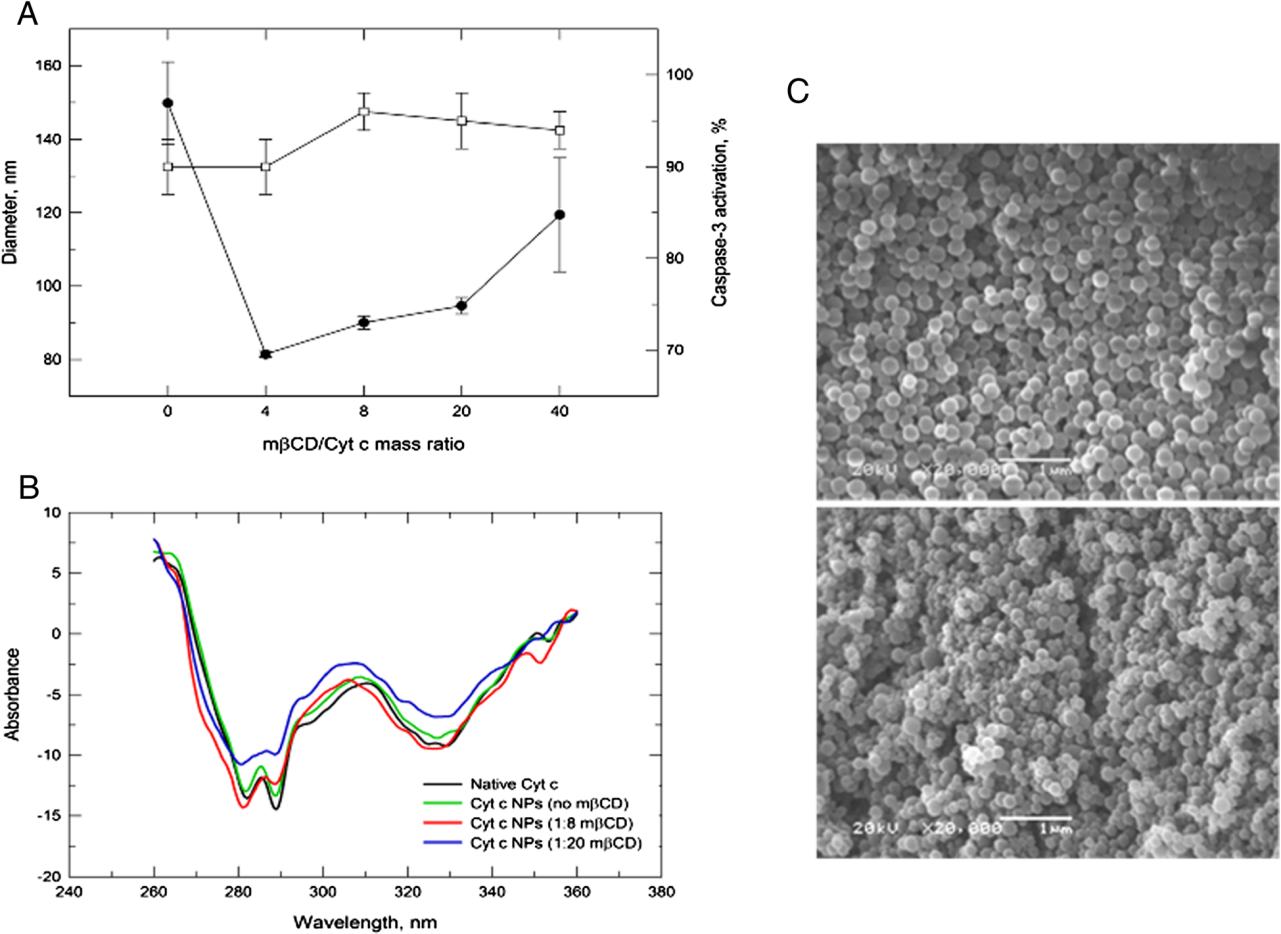
**The effect of methyl-*β*-cyclodextrin (m*β*CD) during the protein precipitation. (A)** Effect of m*β*CD on the particle diameter (─●─) and caspase-3 activation (─□─) after Cyt c nanoprecipitation. **(B)** Near-UV CD spectrum of Cyt c nanoprecipitated in absence or presence of mβCD. **(C)** SEM image of Cyt c NPs made without mβCD (upper picture), and at a 1:8 Cyt c-to-mβCD mass ratio (lower image).

The capability of Cyt c to still interact with Apoptotic Protease Activating Factor 1 (Apaf-1) and induce apoptosis after nanoprecipitation was verified next. Since Cyt c is a cell membrane impermeable protein the experiment was conducted in a cell-free system. The addition of Cyt c to fresh cytosol produces caspase activation [15]. Thus, the integrity of the soluble protein after the nanoprecipitation procedure was compared with native Cyt c and was found to be around 90%. We tested the use of the excipient methyl-β-cyclodextrin (mβCD) to further increase the Cyt c integrity during the nanoprecipitation procedure (Figure 3). The excipient mβCD has been used before to improve enzyme activity in organic solvents [24]. The excipient was co-dissolved with the protein previous to the solvent precipitation step with acetonitrile. Because mβCD is soluble in acetonitrile, the excipient was removed by repeated centrifugation/washing cycles. Our results show that the excipient further improved bioactivity to 96% and decreased the particle diameter to 90 nm.

The impact of the nanoprecipitation procedure on Cyt c tertiary structure was investigated by circular dichroism (CD) measurements after dissolving the NPs in buffer (Figure 3B). The near-UV CD spectra show that the nanoprecipitation procedure did not cause irreversible changes in the protein conformation. The two minima at 286 and 293 nm, characteristic of native Cyt c [25], were present in all NP formulations. However, these two minima were somewhat reduced when mβCD was used at the very high 1:20 mass ratio during nanoprecipitation. Since these two minima correspond to Trp-59 [15] and cyclodextrins have the ability to sequester hydrophobic moieties on protein surfaces [26] it is possible that Cyt c was somewhat destabilized by the additive under these conditions. Such destabilization has been reported for some proteins under aqueous and non-aqueous conditions [24],[27].

Scanning electron microscopy (SEM) was performed to investigate the shape of the NPs (Figure 3C). The SEM images of lyophilized Cyt c NP formulations show that the powder particles obtained had a spherical shape and confirm that the particle size was in the nanometer range.

Above findings demonstrate that the nanoprecipitation method is useful in obtaining nano-scale Cyt c without introducing irreversible functional loss. We also demonstrated that the protein was still able to interact with Apaf-1 to induce apoptosis upon rehydration.

### Surface modification of Cyt c NPs

**Figure 4:**
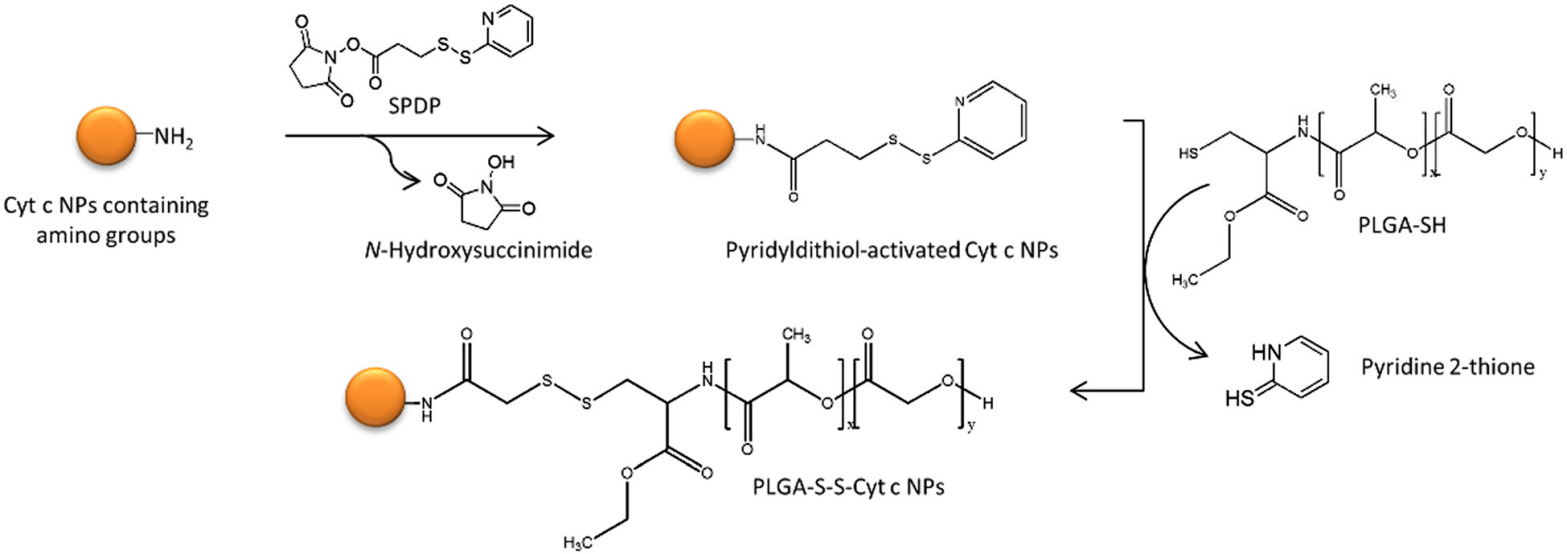
Scheme of the surface modification of Cyt c NPs with PLGA-SH by using a linker.

**Figure 5:**
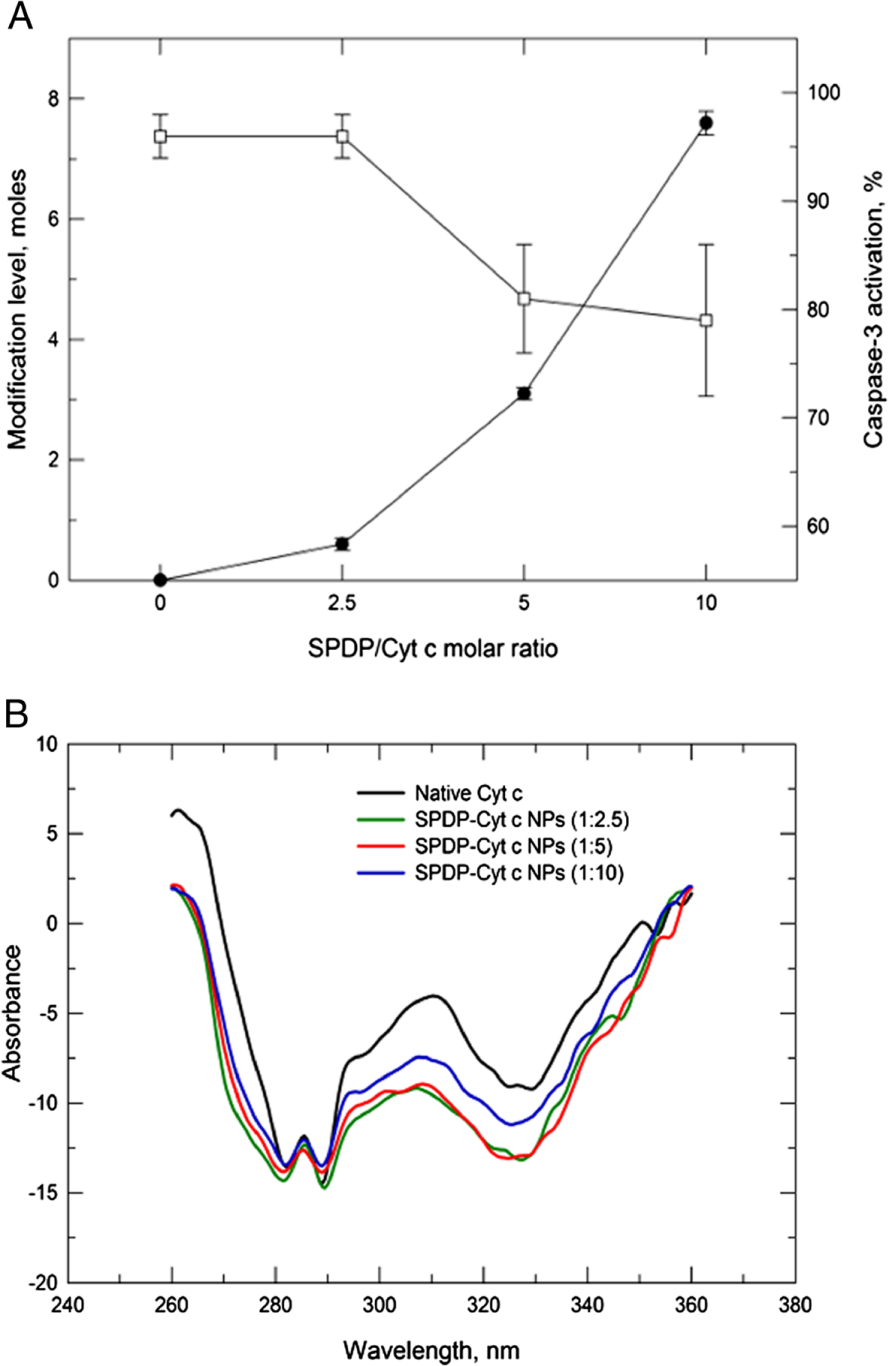
**Characterization of the modification of Cyt c NP surface with the SPDP linker. (A)** SPDP modification level accomplished using increasing reagent concentrations during synthesis (closed symbols) and effect of the modification on *in vitro* caspase-3 activation in a cell-free system (open symbols). **(B)** Near-UV CD spectra of dissolved Cyt c NPs after modification with SPDP.

To make the nanoparticles useful for delivery purposes we proceeded to modify their surface using a two-step procedure (Figure 4). First, the NPs’ surface was modified with the linker SPDP to allow for their coating with PLGA-SH. The level of SPDP modification was determined by measuring the release of pyridine-2-thione at 343 nm after addition of dithiothreitol (DTT) to the reaction products. An increasing amount of linker was attached to the NPs at increasing reagent concentration during the reaction (Figure 5A). Since some of the target amino groups of Cyt c overlap with the Cyt c-Apaf 1 interaction site, increased levels of SPDP linked to Cyt c NPs reduced the ability of Cyt c to interact with Apaf-1 by about 20% (Figure 5A). However, since a large amount of Cyt c should be delivered to each single target cell in the end (a complete NP with roughly over 100,000 molecules), this was acceptable for our application. Circular dichroism (CD) spectra showed that no major changes in Cyt c tertiary structure occurred upon modification with SPDP (Figure 5B). We selected the 5 fold-SPDP modification level for PLGA attachment to have sufficient polymer attachment points.

Next, the PLGA polymer was attached to the linker-modified (activated) NPs in order to prevent them to dissolve in aqueous media (e.g., upon reconstitution in buffer). The level of modification accomplished with PLGA-SH was determined by spectrophotometric analysis of the 2-pyridyldithio group released upon synthesis (Figure 4) at 343 nm. The level of modification of the NPs with PLGA was determined to be 2.06 ± 0.04 moles per mol of Cyt c under the conditions employed (see Methods Section for details). This construct is referred to from here on as PLGA-S-S-Cyt c NPs.

**Figure 6:**
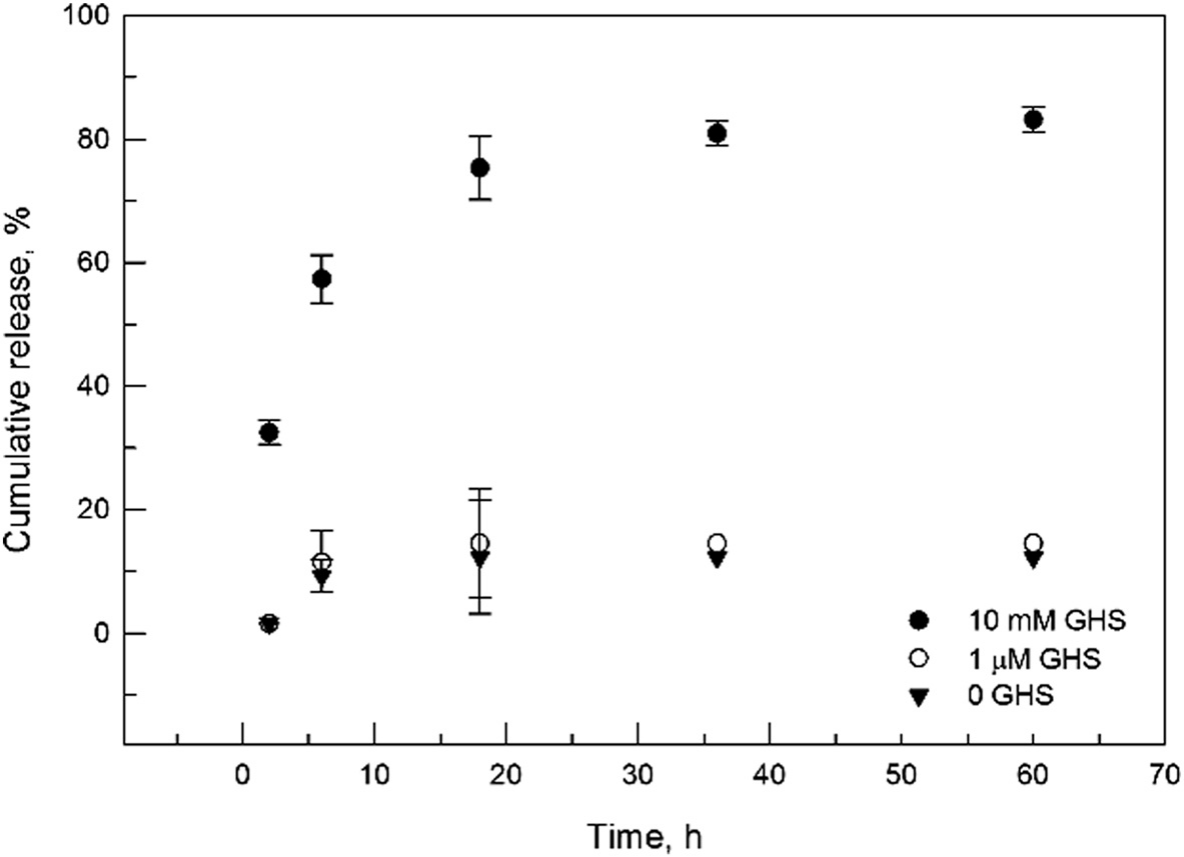
Sustained release of Cyt c from PLGA-coated NPs after exposure to various conditions.

Finally, we verified whether the constructs obtained would behave in the manner we anticipated – stable in aqueous medium under oxidizing conditions and dissolving after reduction-induced polymer shedding under intracellular conditions. PLGA-S-S-Cyt c NPs were placed in release buffer using glutathione concentrations of 0, 1 μM, and 10 mM and incubated at 37°C simulating extra- and intracellular conditions [28]. At pre-determined times the supernatant was removed and the concentration of dissolved Cyt c determined by measuring the heme absorbance at 530 nm. The supernatant was replaced to maintain sink conditions. The concentration of the released protein was used to construct cumulative release profiles (Figure 6).

The PLGA coated Cyt c NPs released ca. 10% of Cyt c during the experiment, most of it during the first few hours as a “burst release”. The amount of burst compares well with most conventional PLGA-based sustained release devices. Release of >80% of Cyt c was accomplished under reducing conditions demonstrating that the system design idea proved correct.

### PLGA-S-S-Cyt c NPs cytotoxic effects to HeLa cells

**Figure 7:**
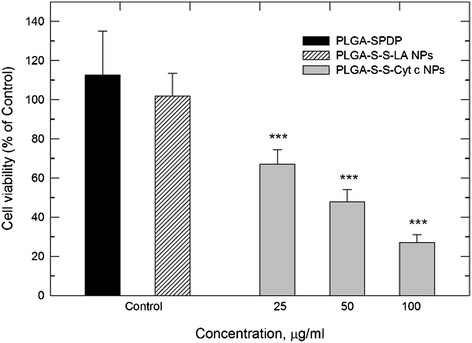
**Viability of HeLa cells treated with PLGA-S-S-Cyt c NPs after 6 h of incubation.** Two controls were included (PLGA-SPDP, PLGA-S-S-LA NPs) and had no effect on HeLa cell viability. Asterisk (***) indicates statistical significance (*p* < 0.001).

To investigate whether the synthesized NPs would display the desired effect on cancer cells, HeLa cells were incubated with PLGA-S-S-Cyt c NPs at 25, 50, and 100 μg/ml Cyt c concentration for 6 h. Cyt c NPs coated with PLGA induced a significant reduction in cell viability after 6 h of incubation, in particular at the 100 μg/ml Cyt c concentration (Figure 7). Several control experiments were performed. PLGA was conjugated to the SPDP linker and added to HeLa cells at the same concentration as in the corresponding experiments with the highest PLGA-S-S-Cyt c NPs concentration. No significant cytotoxicity was observed after 6 h (Figure 7) and even 24 h of incubation (data not shown) with PLGA-SPDP. To further confirm that the cell death after treatment with PLGA-S-S-Cyt c NPs was due to Cyt c, we constructed the same PLGA coated NPs delivery using the non-apoptotic protein α-lactalbumin (LA). The LA-based NPs had no effect on cell viability excluding cytotoxic effects of the delivery system *per se* (Figure 7).

Similar to us, Zhao and coworkers designed a smart nanoparticulate DDS for an apoptotic protein (i.e., caspase-3). The protein was released mediated by intracellular reducing conditions. The study showed evidence of cell death after 48 h of incubation [18] in contrast to 6 h for our DDS.

The DDS designed by us herein also proved much more efficient in killing HeLa cells than our previous smart DDS based on silica NPs [15]. Making the drug Cyt c itself the nanoparticulate delivery system eliminated the main problem encountered with the other system, namely, increasing silica toxicity when trying to deliver sufficient Cyt c to the target cells. Similarly, NPs using other materials [29] could present similar issues as our silica-based delivery system.

Another potential advantage of the newly designed DDS is its negative charge. Although positively charged NPs have shown good cell internalization properties [18],[30],[31], they potentially bind to vascular endothelial cells reducing their tumor accumulation by means of the EPR effect (i.e., the vascular endothelial luminal surface is known to be negatively charged) [2]. In contrast, PLGA has a negative charge at physiological and alkaline pH and it has been demonstrated that it can be internalized by the cell and is able to escape from the endosomes [32]. Therefore, our protein-based NPs could be an alternative to the intracellular delivery also of other apoptotic proteins.

### Investigation of apoptosis induction in HeLa cells by the intracellular release of Cyt c

**Figure 8:**
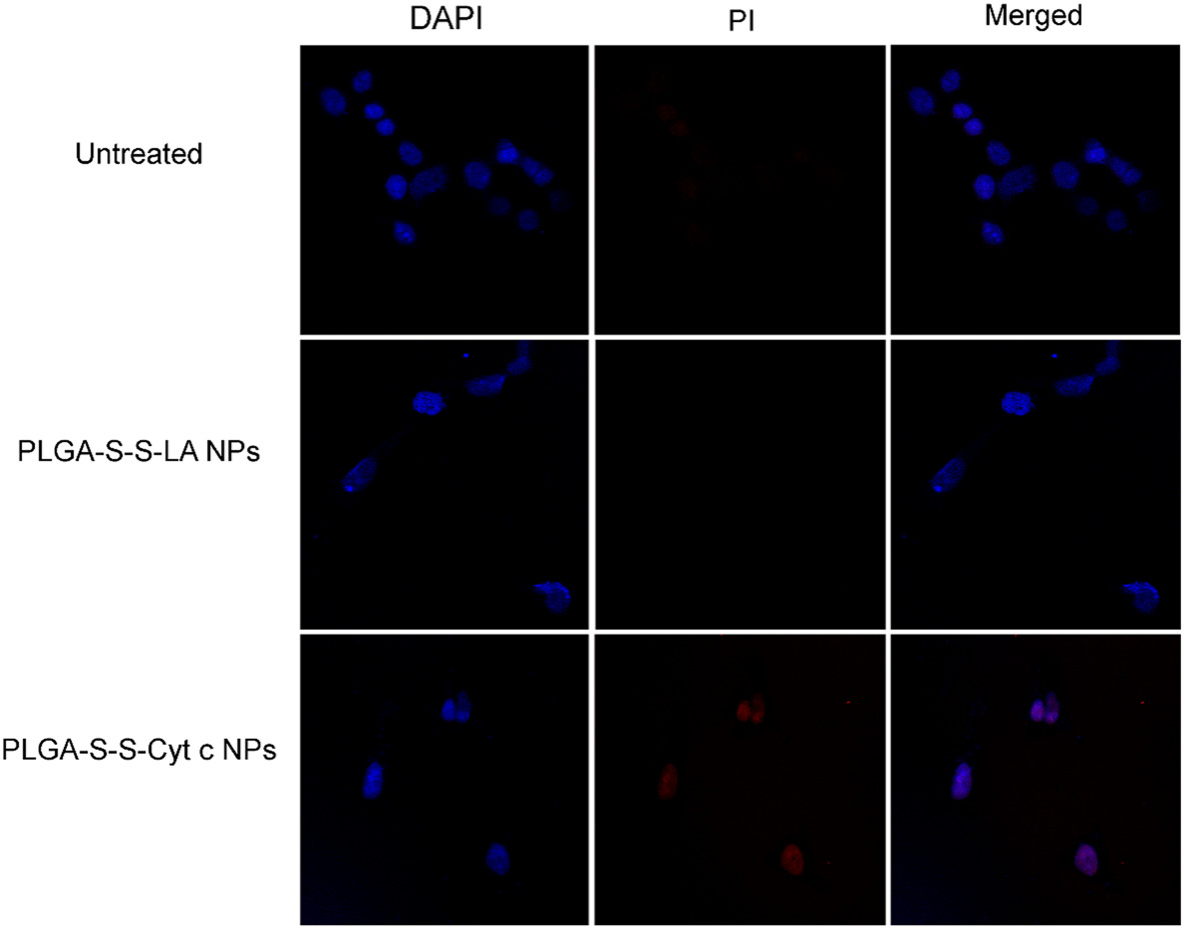
**Study of DAPI and propidium iodine (PI) colocalization, for the detection of apoptotic cells.** Selective induction of apoptosis observed in HeLa cells incubated with the PLGA-S-S-Cyt c NPs. No cellular apoptosis observed in untreated HeLa cells or when incubated with PLGA-S-SLA NPs.

To confirm that the Cyt c-induced cell death observed in the cell viability experiments was indeed due to apoptosis, we assessed the occurrence of nuclear segmentation and chromatin condensation. After HeLa cells were incubated with PLGA-S-S-Cyt c NPs or the PLGA-S-S-LA NPs control for 6 h, the cells were stained with PI and DAPI. Co-localization of DAPI and PI occurred when cells were incubated with Cyt c-based NPs (Figure 8), which points toward nuclear fragmentation and chromatin condensation in the cells indicative of ongoing apoptosis. In contrast, cells without treatment or treated with the PLGA-S-S-LA control showed no indication for dye co-localization and thus apoptosis. These results are in agreement with confocal results by Santra et al [7] who demonstrated that the delivery of Cyt c to the cytoplasm of the cancer cells results in the induction of apoptosis of such cells [7].

## Conclusions

Therapeutic proteins have enormous potential in the treatment and prevention of human diseases but are of limited use because they frequently display low physicochemical stability. Specifically, when injected proteins usually quickly degrade and have short blood half-lives. Thus, the development of DDS able to protect the protein payload from degradation and in addition enabling targeted and controlled delivery is of considerable interest. Herein, we presented a new method for the delivery of Cyt c, an apoptotic protein, to model cancer cells. The main advantage of the system is that the DDS core consisted of Cyt c NPs and thus the drug itself thus overcoming payload limitations of drug-loaded systems. In order to stabilize the protein NPs we coated them with hydrophobic PLGA using a redox-sensitive linker. Only little Cyt c was released under oxidizing conditions, but 80% was released within hours under intracellular conditions. We furthermore demonstrated that PLGA-S-S-Cyt c NPs were able to induce apoptosis in a human cancer cell line while PLGA-S-S-LA NPs as control did not. Redox-responsive polymer-coated protein-based NPs are a simple and effective method for intracellular delivery of Cyt c for induction of apoptosis. We foresee that this system could potentially be used for the delivery of other proteins. However, we like to point out that our system was not tested in a non-cancerous cell line at this point in time because it does not include targeting ligands. Thus, it would probably show comparable toxicity in cancer and non-cancer cell lines since the EPR effect afforded by the nanoparticle formulation would not be relevant *in vitro* studies. It is also important to acknowledge that particle size is by far not the sole determinant of tumor specificity in *in vivo* studies. For example, immune cells can consume the drug delivery system and some tumors maybe very dense in the core disabling EPR-mediated targeting. To address such questions, experiments ongoing in our laboratory focus on *in vivo* experiments using animal models. Tumor selectivity and internalization kinetics and mechanism of PLGA-S-S-Cyt c NPs are being scrutinized as well as the decoration of the system with tumor specific ligands. Combining passive targeting with additional ligand-mediating targeting should not only amplify the specificity of the NPs but also facilitate their efficient cellular uptake.

## Experimental procedures

Cytochrome c from equine heart, reduced glutathione ethyl ester, and a protease inhibitor cocktail were from Sigma-Aldrich (St. Louis, MO). Acetonitrile (HPLC grade) was from Fisher (Waltham, MA). Succinimidyl-3-(2-pyridyldithio) propionate (SPDP) was from Proteochem (Denver, CO). Poly (lactide-*co*-glycolide)-SH with a thiol end cap (PLGA-SH, 30,000 Da, copolymer ratio 1:1) was from Akina, Inc (West Lafayette, IN). 4’, 6-Diamidino-2-phenylindole (DAPI) and propidium iodide (PI) were purchased from Invitrogen (Grand Island, NY). All the reagents were used without further purification. All other chemicals were from various commercial suppliers and were at least of analytical grade. HeLa cells were purchased from the American Type Culture Collection (Manassas, VA) and grown according to the vendor’s instruction.

### Protein nanoprecipitation

Protein nanoparticles were obtained using a similar method as described previously by us [14]. Briefly, Cyt c was solvent-precipitated from nanopure water in presence of the excipient methyl-β-cyclodextrin by adding acetonitrile at a 1:4 volume ratio. Different protein concentrations (5, 10, 20, 40 mg/ml) as well as different mass ratios of protein-to-excipient (1:4, 1:8, 1:20 and 1:40, w/w) were studied. The protein suspension obtained was centrifuged at 6,000 rpm for 15 min, the supernatant discarded, and the pellet vacuum dried for 30 min.

### Determination of the nanoprecipitation yield

Protein concentration and amount of protein aggregates in Cyt c NPs were determined as described by us in detail [33]. In brief, the Cyt c NPs were suspended in 2 ml of potassium phosphate (PBS) buffer for 2 h to dissolve the buffer-soluble fraction. The samples were then subjected to centrifugation at 6,000 rpm for 15 min and the supernatant used to determine the protein concentration. Next, 1 ml of 6 M urea was added to the pellet to dissolve the buffer-insoluble protein fraction. The protein concentration was determined by quantitative ultraviolet (UV) spectrophotometric analysis at 280 nm and also from the heme absorbance at 408 nm. The precipitation yield was calculated from the actual and theoretical quantity of protein recovered after nanoprecipitation and rehydration (%w/w). Cyt c as obtained from the supplier in PBS or 6 M urea was used to construct a calibration curve. The experiments were performed in triplicate, the results averaged, and the standard deviations calculated.

### Synthesis of SPDP-Cyt c NPs

Following the Cyt c nanoprecipitation, the SPDP linker was added directly to the resulting suspension (i.e., in 80% acetonitrile) to accomplish the NPs surface modification with the linker. Different Cyt c-to-SPDP molar ratios (1:2.5, 1:5, 1:10) were tested. After letting the mixture react for 30 min, the suspension was used to determinate the level of modification with SPDP. To stop the reaction and to remove unreacted SPDP and ***N***-hydroxysuccinimide by-products, repeated centrifugation (6,000 rpm, 15 min)/washing cycles were performed. The pyridyldithiol-activated Cyt c (SPDP-Cyt c) NPs pellet was saved for further reaction and analysis. The supernatant was used to determine the concentration of unreacted SPDP by measuring the release of pyridine-2-thione at 343 nm after addition of 10 μL of 15 mg/ml DTT (the absorbance of the supernatant before reaction with DTT was used as blank). The amount of covalent modification of the nanoparticles with the linker was determined from the difference between the initial SPDP concentration and the unreacted SPDP. The experiments were performed in triplicate, the results averaged, and the standard deviations calculated.

### Synthesis of PLGA-Cyt c NPs

HS-PLGA (50 mg) dissolved into 5 ml of Acetonitrile was added to SPDP-Cyt c NPs (5 mg) and reacted at room temperature for 18 h. After reaction, unreacted HS-PLGA and pyridine 2-thione by-products were removed by centrifugation at 6,000 rpm for 10 min. The supernatant was used to determine the level of modification by measuring the concentration of pyridine-2-thione at 343 nm.

### In vitro release of Cyt c

The release of Cyt c from PLGA-S-S-Cyt c NPs was measured similarly as described by us [28]. Briefly, 0.25 mg of PLGA-S-S-Cyt c NPs powder were suspended by sonication in 1 ml of 50 mM PBS with 1 mM EDTA at pH 7.4 and glutathione (GHS) concentrations of 0, 0.001, and 10 mM. Incubation was performed for various times at 37°C, and the NPs were pelleted by centrifugation at 14,000 rpm for 10 min. The supernatant was removed and used to determine the concentration of released Cyt c. The pellet was resuspended in GHS-PBS buffer. The amount of protein released was used to construct cumulative release profiles. The experiments were performed in triplicate, the results averaged, and the standard deviations calculated.

### Dynamic light scattering

Particle sizes of the different formulations of Cyt c NPs were determined by Dynamic light scattering using a DynaPro Titan. The samples were dispersed in DMF and subjected to ultrasonication at 240 W for 30 sec prior to the measurements.

### Scanning electron microscope (SEM)

SEM of the different formulations of Cyt c NPs was performed using a JEOL 5800LV scanning electron microscope at 20 kV. The samples were coated with gold for 10 sec to a thinkness of 10 nm using a Denton Vacuum DV-502A.

### Circular dichroism (CD) spectroscopy

CD spectra were recorded using a JASCO J-1500 High Performance CD spectrometer at room temperature. The protein (Cyt c, Cyt c NPs, or SPDP-Cyt c NPs) was dissolved in nanopure water. CD spectra were acquired from 260 to 350 nm (tertiary structure) at a concentration of 1 mg/ml using a 10 mm quartz cuvette. Each spectrum was obtained by averaging two scans. Spectra of nanopure water blanks were measured prior to the samples and subtracted from the sample spectra.

### Cell-free caspase-3 assay

The cell lysate was obtained as described by us [15]. Briefly, the cell-free reaction was initiated by adding Cyt c or the different Cyt c NP formulations (e.g. 100 μg/mL of Cyt c NPs or SPDP-Cyt c NPs) to freshly purified cytosol (3 mg/ml) in a total reaction volume of 100 μL. The reaction was incubated at 37°C for 150 min. Afterwards the caspase-3 assay was performed following the manufacturer’s protocol (CaspACE™ assay; Promega, Madison, WI). The plate was incubated overnight at room temperature and the absorbance at 405 nm was measured in each well using a Thermo Scientific Multiskan FC. All measurements were performed in triplicate.

### Cell culture

HeLa cells were maintained in accordance with the ATCC protocol. Briefly, the cells were cultured in minimum essential medium (MEM) containing 1% L-glutamine, 10% fetal bovine serum (FBS), and 1% penicillin in a humidified incubator with 5% CO_2_ and 95% air at 37°C. All experiments were conducted before cells reached 25 passages. For the cell viability and confocal microscopy experiments, HeLa cells were seeded in 96-well plates or chambered cover-slides (4 wells), respectively, for 24 h in MEM containing 1% L-glutamine, 10% FBS, and 1% penicillin. Subsequently, cell growth was arrested by decreasing the FBS concentration in the medium to 1% for 18 h. Then, cells were exposed and incubated with the different bioconjugates for 6 h.

### Cell viability assay

Mitochondrial function was measured using the CellTiter 96 aqueous non-radioactive cell proliferation assay from Promega Corporation. HeLa cells (5,000 cells/well) were seeded in 96-well plates as described above. Cells were incubated with serial dilutions of PLGA-S-S-Cyt c NPs (25, 50 and 100 μg/mL of Cyt c) for 6 h. Controls, such as, PLGA-SPDP (80 μg/mL) and NPs of a non-apoptotic protein (i.e. α-lactalbumin at 100 μg/mL) were also tested. After incubation, 20 μL of 3-(4, 5-dimethylthiazol-2-yl)-5-(3-carboxymethoxyphenyl)-2-(4-sulfophenyl)-2H-tetrazolium, inner salt (MTS) and phenazine methosulfate (PMS) was added to each well (333 μg/mL MTS and 25 μM PMS). After 1 h, the absorbance at 492 nm was measured using a microplate reader. HeLa cells treated with 2 μM staurosporin for 6 h were used as positive control and cells without treatment were used as negative control. The relative cell viability (%) was calculated by:

(1) $$\mathrm{Relative}\;\mathrm{cell}\;\mathrm{viability}\;\left(\%\right)=\frac{\mathrm{Abs}\;\mathrm{test}\;\mathrm{sample}}{\mathrm{Abs}\;\mathrm{control}}\times \;100$$

*T*-test analysis was used for comparison of two independent groups for cell viability. Difference between control (untreated cells) and experimental groups (i.e., PLGA-SPDP, PLGA-S-S-LA, NPs PLGA-S-S-Cyt c NPs) was considered statistically significant at p < 0.05.

### Investigation of apoptosis induction in HeLa cells by the intracellular release of Cyt c

HeLa cells (25,000 cells) were seeded in chambered cover-glass (4-wells) as previously described by us [15]. The cells were incubated with PLGA-S-S-Cyt c NPs at a 25 μg/mL Cyt c concentration at 37°C for 6 h. For detection of apoptosis-dependent nuclear fragmentation, the cells were washed with PBS (1X) and incubated initially with DAPI (300 nM) and thereafter with PI (75 μM) for 5 min each. HeLa cells were then fixed using 3.7% formaldehyde. The coverslips were examined under a Zeiss laser-scanning microscope 510 using a 67× objective. Co-localization of DAPI and PI upon internalization into HeLa cells was determined, which is representative of highly condensed and fragmented chromatin in apoptotic cells [7],[34],[35]. DAPI was excited at 405 nm and its emission was detected at 420-480 nm. PI was excited at 561 nm and was detected above 600-674 nm.

## Abbreviations

Cyt c: Cytochrome c

Apaf-1: Apoptotic protease activating factor 1

LA: α-lactalbumin

PLGA: Poly (lactic-co-glycolic) acid

EPR: Enhanced permeability and retention

NPs: Nanoparticles

DDS: Drug delivery system

MSN: Mesoporous silica nanoparticles

MEM: Minimum essential medium

FBS: Fetal bovine serum

DAPI: 4’,6-diamidino-2-phenylindole

PI: Propidium iodine

MTS: 3-(4, 5-dimethylthiazol-2-yl)-5-(3-carboxymethoxyphenyl)-2-(4-sulfophenyl)-2H-tetrazolium, inner salt

PMS: Phenazine methosulfate

GHS: Glutathione

SPDP: Succinimidyl-3-(2-pyridyldithio) propionate

mβCD: Methyl-β-cyclodextrin

DTT: Dithiothreitol

EDTA: Ethylenediaminetetraacetic acid

SEM: Scanning electron microscopy

CD: Circular dichroism

PBS: Phosphate buffer solution

## Competing interests

The authors declare they have no competing interests.

## Authors’ contributions

MMC carried out all the experimental studies, contributed in the design of the study, analyzed data, and drafted the manuscript. CMF participated in the caspase-3 and cell viability assays, and helped drafting the manuscript. TGR participated in the nanoprecipitation and nanoparticle surface modification studies. YDR participated in the circular dichroism spectroscopy and the dynamic light scattering studies. AM participated in the dynamic light scattering and confocal studies. JM participated in the experimental design of cell culture experiments. MM performed the statistic analysis. KG conceived the study, participated in its design and coordination, and finalized the manuscript. All authors read and approved the final manuscript.

## References

[B1] Danhier F Feron O Preat V To exploit the tumor microenviroment: passive and active tumor targeting of nanocarriers for anti-cancer drug delivery J Control Release 2010 148 135 146 10.1016/j.jconrel.2010.08.027 20797419

[B2] Fang J Nakamura H Maeda H The EPR effect: unique features of tumor blood vessels for drug delivery, factors involved, and limitations and augmentation of the effect Adv Drug Deliv Rev 2011 63 136 151 10.1016/j.addr.2010.04.009 20441782

[B3] Morachis JM Mahmoud EA Almutairi A Physical and chemical strategies for therapeutic delivery by using polymeric nanoparticles Pharmacol Rev 2012 64 505 519 10.1124/pr.111.005363 22544864 PMC3400833

[B4] Morigi V Tocchio A Bellavite-Pellegrini C Sakamoto JH Arnone M Tasciotti E Nanotechnology in medicine: from inception to market domination J Drug Deliv 2012 2012 1 7 10.1155/2012/389485 PMC3312282 22506121

[B5] Lanni JS Lowe SW Licitra EJ Liu JO Jacks T p53-independent apoptosis induced by paclitaxel through an indirect mechanism Proc Natl Acad Sci U S A 1997 94 9679 9683 10.1073/pnas.94.18.9679 9275183 PMC23249

[B6] Ryan KM Phillips AC Vousden KH Regulation and function of the p53 tumor suppressor protein Curr Opin Cell Biol 2001 13 332 337 10.1016/S0955-0674(00)00216-7 11343904

[B7] Santra S Kaittanis C Perez JM Cytochrome C encapsulating theranostic nanoparticles: a novel bifunctional system for targeted delivery of therapeutic membrane-impermeable proteins to tumors and imaging of cancer therapy Mol Pharm 2010 7 1209 1222 10.1021/mp100043h 20536259 PMC2914151

[B8] Yamada Y Harashima H Mitochondrial drug delivery systems for macromolecule and their therapeutic application to mitochondrial diseases Adv Drug Deliv Rev 2008 60 1439 1462 10.1016/j.addr.2008.04.016 18655816

[B9] Solá RJ Griebenow K Effects of glycosylation on the stability of protein pharmaceuticals J Pharm Sci 2009 98 1223 1245 10.1002/jps.21504 18661536 PMC2649977

[B10] Manning MC Chou DK Murphy BM Payne RW Katayama DS Stability of protein pharmaceuticals: an update Pharm Res 2010 27 544 575 10.1007/s11095-009-0045-6 20143256

[B11] Brown LR Commercial challenges of protein drug delivery Expert Opin Drug Deliv 2005 2 29 42 10.1517/17425247.2.1.29 16296733

[B12] Slowing II Trewyn BG Lin VSY Mesoporous silica nanoparticles for intracellular delivery of membrane-impermeable proteins J Am Chem Soc 2007 129 8845 8849 10.1021/ja0719780 17589996

[B13] Solaro R Targeted delivery of protein drugs by nanocarriers Materials 2010 3 1928 1980 10.3390/ma3031928

[B14] Morales-Cruz M Flores-Fernández GM Morales-Cruz M Orellano EA Rodriguez-Martinez JA Ruiz M Griebenow K Two-step nanoprecipitation for the production of protein-loaded PLGA nanospheres Results Pharma Sci 2012 2 79 85 10.1016/j.rinphs.2012.11.001 23316451 PMC3541529

[B15] Méndez J Morales-Cruz M Delgado Y Figueroa CM Orellano EA Morales M Monteagudo A Griebenow K Delivery of chemically glycosylated cytochrome c immobilized in mesoporous silica nanoparticles induces apoptosis in hela cancer cells Mol Pharm 2014 11 102 111 10.1021/mp400400j 24294910 PMC3905321

[B16] Langer K Balthasar S Vogel V Dinauer N von Briesen H Schubert D Optimization of the preparation process for human serum albumin (HSA) nanoparticles Int J Pharm 2003 257 169 180 10.1016/S0378-5173(03)00134-0 12711172

[B17] Jahanshahi M Babaei Z Protein nanoparticle: a unique system as drug delivery vehicles Afr J Biotechnol 2008 7 4926 4934

[B18] Zhao M Biswas A Hu B Joo K Wang P Gu Z Tang Y Redox-responsive nanocapsules for intracellular protein delivery Biomaterials 2011 32 5223 5230 10.1016/j.biomaterials.2011.03.060 21514660

[B19] Shive M Anderson J Biodegradation and biocompatibility of PLA and PLGA microspheres Adv Drug Deliv Rev 1997 28 5 24 10.1016/S0169-409X(97)00048-3 10837562

[B20] Lü JM Wang X Marin-Muller C Wang H Lin PH Yao Q Chen C Current advances in research and clinical applications of PLGA based nanotechnology Expert Rev Mol Diagn 2009 4 325 341 10.1586/erm.09.15 19435455 PMC2701163

[B21] Nitta SK Numata K Biopolymer-based nanoparticles for drug/gene delivery and tissue engineering Int J Mol Sci 2013 14 1629 1654 10.3390/ijms14011629 23344060 PMC3565338

[B22] Torchilin V Tumor delivery of macromolecular drugs based on the EPR effect Adv Drug Deliv Rev 2011 63 131 135 10.1016/j.addr.2010.03.011 20304019

[B23] Kim KY Nanotechnology platforms and physiological challenges for cancer therapeutics Nanomedicine 2007 3 103 110 10.1016/j.nano.2006.12.002 17442621

[B24] Griebenow K Diaz-Laureano Y Santos AM Montañez-Clemente I Rodriguez L Vidal M Barletta G Improved enzyme activity and enantioselectivity in organic solvents by methyl-β-cyclodextrin J Am Chem Soc 1999 121 8157 8163 10.1021/ja990515u

[B25] Davies AM Guillemette JG Smith M Greenwood C Thurgood AG Mauk AG Moore GR Redesign of the interior hydrophilic region of mitochondrial cytochrome c by site-directed mutagenesis Biochemistry 1993 32 5431 5435 10.1021/bi00071a019 8388720

[B26] Aachmann FL Otzen DE Larsen KL Wimmer L Structural background of cyclodextrin–protein interactions Protein Eng 2003 16 905 912 10.1093/protein/gzg137 14983070

[B27] Cooper A Effect of cyclodextrins on the thermal stability of globular proteins J Am Chem Soc 1992 114 9208 9209 10.1021/ja00049a074

[B28] Méndez J Monteagudo A Griebenow K Stimulus-responsive controlled release system by covalent immobilization of an enzyme into mesoporous silica nanoparticles Bioconjug Chem 2012 23 698 704 10.1021/bc200301a 22375899 PMC3329583

[B29] Besaratinia A Pfeifer GP A review of mechanisms of acrylamide carcinogenicity Carcinogenesis 2007 28 519 528 10.1093/carcin/bgm006 17234719

[B30] Yu B Zhang Y Zheng W Fan C Chen T Positive surface charge enhances selective cellular uptake and anticancer efficacy of selenium nanoparticles Inorg Chem 2012 51 8956 8963 10.1021/ic301050v 22873404

[B31] Choi SY Jang SH Park J Jeong S Park JH Ock KS Lee K Yang SI Joo SW Ryu PD Lee SY Cellular uptake and cytotoxicity of positively charged chitosan gold nanoparticles in human lung adenocarcinoma cells J Nanopart Res 2012 14 1234 10.1007/s11051-012-1234-5

[B32] Panyam J Zhou WZ Prabha S Sahoo SK Labhasetwar V Rapid endo-lysosomal escape of poly (DL-lactide-co-glycolide) nanoparticles: implications for drug and gene delivery FASEB J 2002 16 1217 1226 10.1096/fj.02-0088com 12153989

[B33] Castellanos IJ Griebenow K Improved α-chymotrypsin stability upon encapsulation in PLGA microspheres by solvent replacement Pharm Res 2003 20 1873 1880 10.1023/B:PHAM.0000003388.59659.fa 14661935

[B34] Bratton SB Salvesen GS Regulation of the apaf-1-caspase-9 apoptosome J Cell Sci 2010 123 3209 3214 10.1242/jcs.073643 20844150 PMC2939798

[B35] Shacter E Williams JA Hinson RM Senturker S Lee YJ Oxidative stress interferes with cancer chemotherapy: inhibition of lymphoma cell apoptosis and phagocytosis Blood 2000 96 307 313 10891466

